# Missing Renal Stone Diagnosis in Dementia Patients With Recurrent Urinary Tract Infections: A Case Report and Literature Review

**DOI:** 10.7759/cureus.58908

**Published:** 2024-04-24

**Authors:** Sakshi Jain, Rashi Bharat Patel, Lovekumar Vala, SudhaRani Kinthada, Neel Patel, Shikha Jain, Tanzina Khan, Athmananda Nanjundappa, Vaishnavi Sirekulam, Nishthaben Naik, Chandu Siripuram, Harmeet Gill

**Affiliations:** 1 Department of Geriatrics, Hackensack University Medical Center, Hackensack, USA; 2 College of Medicine, Tianjin Medical University, Tianjin, CHN; 3 Department of Anatomy, Shantabaa Medical College, Amreli, IND; 4 Department of Obstetrics and Gynecology, Rangaraya Medical College, Kakinada, IND; 5 Department of Medical Education, GMERS Medical College Gotri, Vadodara, IND; 6 Department of Medicine, MVJ Medical College and Research Hospital, Bengaluru, IND; 7 Department of Internal Medicine, Bangladesh Medical College, Dhaka, BGD; 8 Department of Internal Medicine, MedStar Franklin Square Medical Center, Baltimore, USA; 9 Department of Medicine, Vijayanagar Institute of Medical Sciences, Ballari, IND; 10 Department of Health & Family Welfare, Primary Health Center, Bigri, Navsari, IND; 11 Department of Hospital Medicine, Geisinger Medical Center, Scranton, USA; 12 Department of Medicine, HopeHealth, Florence, USA

**Keywords:** urinary tract infection (uti), renal stone, geriatric, elderly, dementia

## Abstract

In older adults, diagnosing, treating, and preventing urinary tract infections (UTIs) can be challenging. This case is of an 82-year-old female of white descent, who was admitted to a post-acute care facility following hospitalization for delirium and a UTI. Hypoactive delirium may be the only clinical manifestation of recurrent UTI. Due to challenges in obtaining a history from this patient with dementia, she had to be admitted multiple times for sepsis. During her final hospitalization, a CT scan of the abdomen and pelvis was ordered, which revealed an obstructed kidney stone as the cause of her recurrent UTIs. Recurrent UTIs especially in patients with dementia should prompt further imaging to look for kidney stones. Factors like dehydration and poor oral intake are risk factors for kidney stones, which patients with dementia are susceptible to.

## Introduction

Recurring urinary tract infection (UTI) poses a considerable health concern, ranking as the second most prevalent infection among residents in nursing homes [1,2]. As per recent evidence, the prevalence of UTI ranges from 0.6% to 21.8%, with an incidence of 0.3 to 0.8 infections per 1000 resident care days [1]. It is estimated that UTIs cost at least US$2-3 billion per year in the United States [3]. The occurrence of UTIs is more prevalent among residents of nursing homes compared to the general population [4]. Recurrent UTIs in this vulnerable population can lead to severe morbidity, increased healthcare utilization, diminished quality of life, low morale, antibiotic resistance, and increased anxiety [5,6]. The diagnosis of UTIs in nursing home patients is challenging because of obstacles in communication, persistent genitourinary symptoms, and the presence of comorbidities. Understanding the causes and factors that lead to recurring UTIs and using effective diagnostic and treatment methods are necessary to improve the health of residents and to reduce the load on healthcare systems [7].

Many studies and guidelines have been developed for diagnosing UTIs in older adults. These include the revised McGreers criteria, which is a well-known protocol used to screen for infections in long-term care settings and help with diagnosis in patients who are cognitively impaired [8]. However, there is a lack of guidance on establishing the etiology of recurrent UTIs, such as kidney stones in patients with cognitive impairment. In high-risk older adults, insufficient oral intake and dehydration are significant factors that can trigger the development of kidney stones. Communication problems and atypical symptoms in this group of patients can make it hard to get a full history, which can cause a missed diagnosis of nephrolithiasis [9]. It is crucial to consistently investigate the root cause of recurrent UTIs, such as obstructive kidney stones, particularly in the presence of relevant risk factors like dehydration.

This case report and literature review provides the clinical course of a nursing home resident with recurrent UTIs, concentrating on key aspects, such as the patient's cognition, clinical presentation, diagnostic evaluation, management, and outcomes. The development of a diagnostic algorithm is suggested to determine the underlying causes of recurrent UTIs, one of them being kidney stones in older adults especially those with dementia.

This article was previously presented as a meeting abstract at the 2023 American Geriatrics Society Scientific Meeting on May 5, 2023.

## Case presentation

A post-acute care facility admitted an 82-year-old female of white descent after her hospitalization for delirium and a UTI. Although she underwent rehabilitation, her limited progress in therapy and concurrent dementia necessitated long-term care. The patient consistently denied pain or dysuria during her hypoactive delirium episodes. She seldom spiked a fever and labs always showed mild leukocytosis. Two episodes were treated with antibiotics at the facility. However, the challenges of obtaining a comprehensive history from this patient with dementia, combined with atypical symptoms, also resulted in multiple hospitalizations for sepsis due to delayed diagnoses over a span of six months.

**Figure 1:**
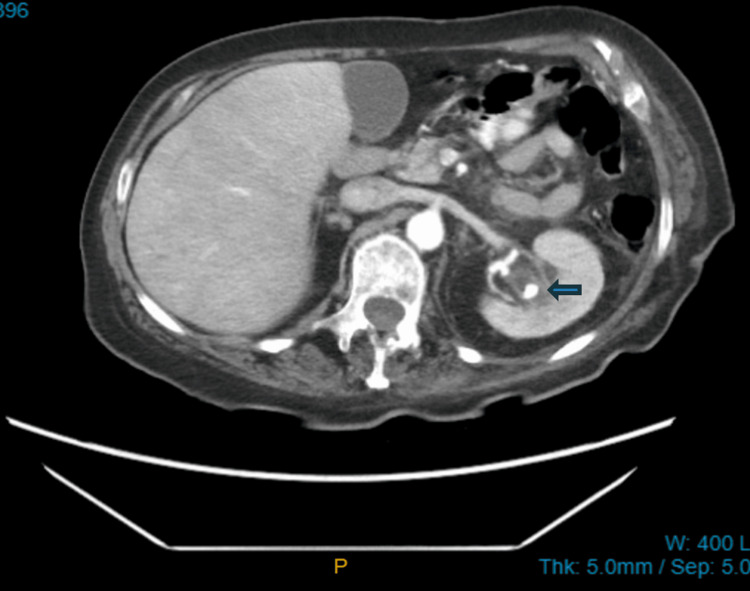
Axial view CT scan abdomen and pelvis showing left-sided nephrolithiasis The blue arrow demonstrates a large calcified stone measuring 11 × 11 × 10.4 cm.

**Figure 2:**
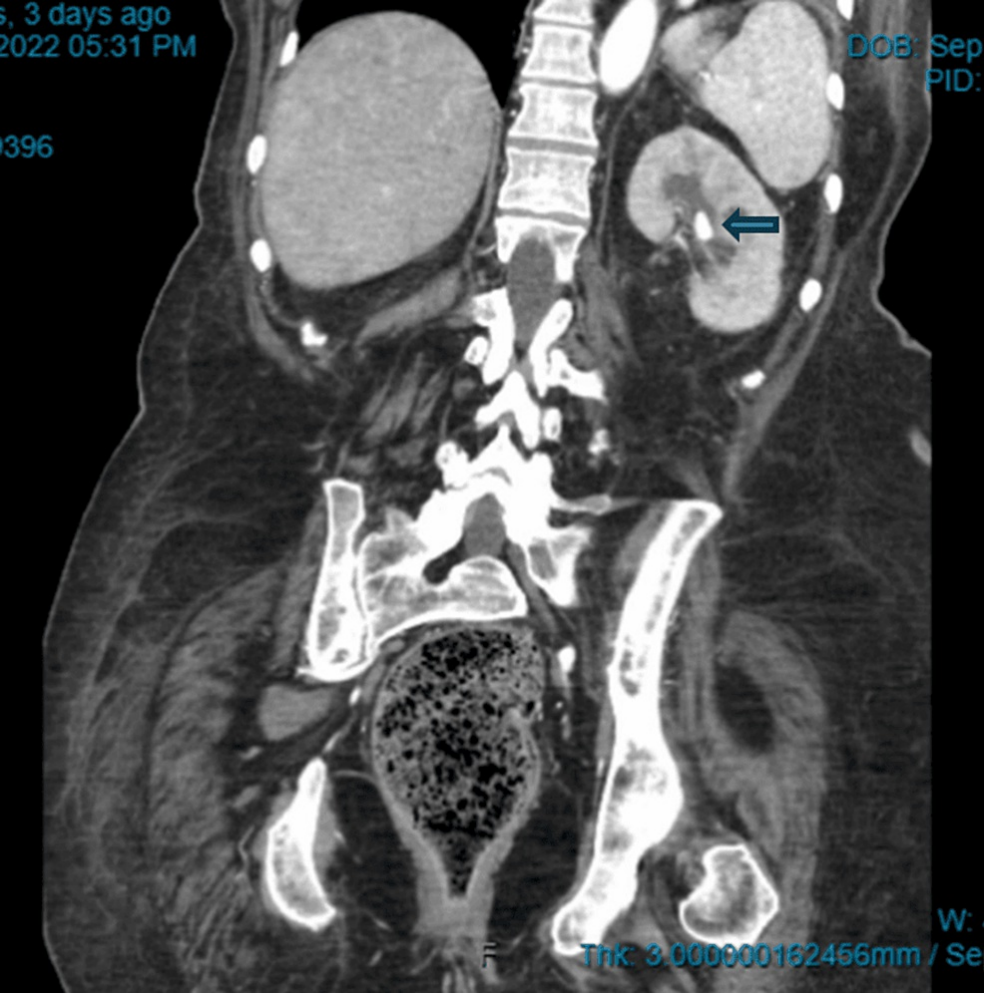
Coronal view CT scan of the abdomen and pelvis showing left-sided nephrolithiasis The blue arrow demonstrates a calcified stone measuring 11 × 11 × 10.4 cm.

UTI diagnosis was made at each hospital admission with a positive urinalysis (UA), urine culture, and leukocytosis seen in her blood work. Each admission involved intravenous antibiotics, intravenous fluids, and a discharge back to the facility. She was hospitalized three times. Her fourth hospitalization for yet another UTI diagnosed with a UA and urine culture prompted further action leading the nursing home attending provider to contact the hospitalist for computed tomography (CT) of the abdomen and pelvis. The CT scan of the abdomen and pelvis revealed a left-sided kidney stone that was obstructed, as shown in Figure 1 and Figure 2. Urology was consulted, and this was managed through cystoscopy and ureteral stent placement. Urology recommended outpatient nephrostomy tube placement, and the patient returned to the nursing home. Post-hospitalization, the patient remained stable with no UTI recurrence, no delirium, and no further hospitalizations for sepsis.

## Discussion

Older adults can present with atypical symptoms of UTI, but not many studies have looked into how common atypical presentations are and what factors are linked to them [9]. The clinical range of UTIs extends from asymptomatic bacteriuria to symptomatic and recurrent cases. In severe instances, it can lead to sepsis necessitating hospitalization [10]. UTIs rank among the most prevalent infections, impacting nearly 50% of women during their lifetime, and about half of these women encounter a recurrence within six to 12 months [11]. One systematic literature review explored the association between recurrent UTIs and kidney stones and found that they can be mutually coexistent when risk factors are present [12]. This case report showcases the diagnostic challenges in patients with cognitive dysfunction and the importance of imaging investigations in assessing the underlying cause of recurrent UTIs.

Since delirium might be the only presenting symptom in older adults, an unreliable history makes diagnosing kidney stones extremely challenging. They might present with acute functional or cognitive decline. Potential explanations for this atypical presentation include the presence of cognitive impairment, such as dementia or delirium, which hinders obtaining a reliable medical history [13]. A systematic review supports the evidence that patients suffering from kidney stone disease often present with recurrent or concomitant urinary tract infections [14]. Table 1 describes previously published case reports with demographics, initial presentation, diagnostic tests, findings, and management of patients with UTI and some with UTI and kidney stones.

**Table 1:** Previously published case reports with demographics, initial presentation, diagnostic tests, findings, and management of patients with UTI and kidney stones KUB: kidney, ureter, and bladder; HbA1c: glycated haemoglobin; CBC: complete blood count; CMP: comprehensive metabolic panel; USG: ultrasound; OD: once a day; CFU/ml: colony-forming unit per milliliter; S.Na: serum sodium; BUN: blood urea nitrogen; CT scan: computed tomography scan; WBCs: white blood cells

Study name	Demographics	Initial presentation	Diagnostic tests performed	Findings	Management/outcomes
Khalid et al., 2022 [14]	A 60-year-old woman	A one-week history of fever with dysuria	KUB, HbA1c, CBC, CMP, urinalysis and urine culture, bladder ultrasound	KUB showed no mass or calculus in the bladder. It showed a simple cyst at the lower pole, measuring 1.9 x 1.8 cm. Lab results: serum creatinine = 1.48 mg/dl, HbA1c = 7.98. Ultrasound of the urinary bladder showed no presence of mass or calculus. Urine culture was positive for >100,000 CFU/ml of *Flavobacterium* species.	Treated for atrophic vaginitis with estrogen cream topically for two weeks and cranberry tablets (475 mg) twice daily for one month. Received intravenous antibiotics piperacillin and tazobactam 4.5 g twice daily for five days. Prescribed fosfomycin 500 mg every eight hours for three months.
Nassour et al., 2013 [15]	A 71-year-old woman	The patient presented with lethargy.	Urinalysis and urine culture	Urine culture with *Escherichia coli* and *Proteus mirabilis*	Treated with intravenous ciprofloxacin for two days. She was discharged back to the nursing home on hospital day 4 with a prescription to take oral ciprofloxacin for three days.
Balouch et al., 2022 [16]	A 91-year-old male	The patient presented with dysuria, urinary frequency, and urgency.	Urinalysis and urine culture	Urinalysis was positive for leukocyte esterase. Urine culture grew *Aerococcus urinae* of 100,000 CFU/mL, along with a smaller quantity of mixed gram-positive organisms.	He was treated with oral nitrofurantoin and subsequently recommended switching to a seven-day course of oral cephalexin.
Mukaya et al., 2016 [17]	An 85-year-old man	A patient with dementia presented with a one-day history of agitation and confusion with complaints of urinary frequency, history of fever, and chills.	CBC, CMP, urinalysis, urine culture, CT scan of the abdomen and pelvis	Laboratory results revealed a creatinine level of 13.6 mg/dL, blood urea nitrogen of 245 mg/dL, potassium of 7.5 mEq/L, bicarbonate level of 17 mEq/L, anion gap of 23 mEq/L, and WBC count of 24,770/µL. Urinalysis revealed 100 white blood cells per high-power field and 3+ blood, although the urine culture result was negative. A CT scan of the abdomen and pelvis indicated a 5-mm calculus at the left vesicoureteric junction causing hydronephrosis and calyceal rupture.	Supportive care was provided, including a Foley catheter, intravenous fluids, and the management of electrolyte imbalances, particularly hyperkalemia.
Hur et al., 2016 [18]	A 60-year-old Korean female	The patient presented with a fever of 38.6 °C accompanied by left-sided flank pain.	CBC, urinalysis, BUN and creatinine, CT scan of the abdomen	WBC was 15.24 × 10^9^/L. Urinalysis showed a pH of 7, absence of nitrates, and 20–29 white blood cells per high-power field. The abdominal CT scan showed a 6-mm obstructive stone in the left renal pelvis, accompanied by left hydroureteronephrosis and thickening of the proximal ureter wall. The urine culture showed methicillin-resistant *S. saprophyticus.*	The patient received intravenous ciprofloxacin, and the condition showed improvement after a seven-day course of ciprofloxacin.
Adhikari et al., 2022 [19]	A 62-year-old female	The patient presented with 12 hours of urinary retention. She had a history of hesitancy at urination, increased frequency, interruption of the urinary stream, urgency, and a feeling of incomplete voiding for the last year. She had been complaining of mild, dull lower abdominal pain for the last six months.	CBC, urinalysis, urine culture, KUB, renal ultrasonography	Urinalysis indicated a very high white blood cell count and few red blood cells. Urine culture showed *Escherichia coli*. A KUB showed a radio-opacity in ​​the pelvic region measuring 9 x 8 cm in size. Renal ultrasonography revealed bilateral mild hydronephrosis with a large bladder stone.	Open cystolithotomy was performed under spinal anesthesia. She was treated with ciprofloxacin for *E. coli*.
Elmanar et al., 2022 [20]	A 53-year-old male	The patient complained of flank pain and had a history of kidney stones with no abdominal pain or fever.	Urinalysis, KUB, abdominal ultrasound, abdominal CT scan	Urinalysis showed hematuria and pyuria. The KUB showed ground-glass opacity at the left upper lower quadrant of the abdomen. Ultrasonography showed severe obstruction with pelvicalyceal system dilatation and echogenic material, suggesting pyelonephrosis. Abdominal CT showed thick fluid filling the left pelvicalyceal system, suggesting pyelonephrosis.	A percutaneous nephrostomy catheter was inserted, followed by a pus culture, resulting in Group B Streptococcus. The patient showed improvement after the administration of antibiotics.
Sandhu et al., 2018 [21]	A 55-year-old male	The patient presented with nausea, vomiting, weakness, reduced urine output, and a pre-syncopal episode.	CBC, CMP, CT scan	The abdominal and pelvic CT scans revealed two obstructive stones, measuring 28 and 30 mm, located in the left proximal ureter, along with one obstructive stone measuring 30 mm in the right midureter.	He was diagnosed with acute renal failure from bilateral ureteral obstruction due to uric acid stones. A treatment of percutaneous chemolysis with potassium citrate and 3 amps (ampoule) of bicarbonate in a 20-cc (cubic centimeter) solution every six hours. By the fifth day of hospitalization, the majority of the stones had resolved, and a follow-up CT scan indicated the presence of only punctate, non-obstructing calculi.
Magro et al., 2019 [22]	A 73-year-old male	The patient came in with a complaint of hematuria. He was seen a week prior for dysuria, urinary urgency, and two episodes of gross hematuria.	CBC, urinalysis, KUB, abdominal ultrasound, CT urogram	Urinalysis showed an acidic pH and the presence of erythrocytes in the urinary sediment, in the absence of nitrites and bacteriuria. The KUB showed bladder distension and several sub and pericentromeric calculi at the level of the medium calyceal group, to the right of the 10 mm stone.	Endoscopic lithotripsy was performed. At two months, it was noted that there were no new episodes of hematuria and improvement in urinary symptoms.
Agrawal et al., 2019 [23]	A 56-year-old female	The patient presented with lower abdominal pain and dysuria for three days. She reported a history of multiple episodes of dysuria that had been treated with unspecified antibiotics for presumed urinary tract infections in the past six months in Mexico.	CBC, CMP, urinalysis, CT scan of the abdomen and pelvis	Laboratory findings revealed a WBC of 13,100 cells/μL with left shift. Urinalysis had positive leukocyte esterase, and there was no bacterial growth in the final urine culture. Non-contrast CT of the abdomen demonstrated a large calcified stone measuring 11 × 11 × 10.4 cm, nearly occluding the urinary bladder with bilateral hydroureter and hydronephrosis.	She was treated with piperacillin-tazobactam and bilateral percutaneous nephrostomy tube placement. On day 3, she underwent open vesicolithotomy and suprapubic tube placement with the removal of the stone. She was discharged due to improvement of kidney function and treated with a 10-day course of amoxicillin-clavulanate.

All case reports are of older adults 50 years and above. All patients in the younger age group between ages 50-69 were able to relay a history and presented with typical signs and symptoms of fever, elevated white blood cell count, dysuria, or flank pain. Two patients in the case reports by Nassour et al. [15] and Mukaya et al. [17] above age 70 presented with atypical symptoms of lethargy, confusion, and agitation. Of these two cases, only the 85-year-old patient with dementia had some typical symptoms of urinary frequency, fever, and chills, which prompted a CT abdomen and pelvis showing a kidney stone. The 71-year-old frail nursing home resident who was unable to provide a reliable history did not receive further imaging despite having risk factors for dehydration and poor oral intake.

Recurrent UTIs in older adults with or without cognitive dysfunction having appropriate risk factors of dehydration should prompt a high degree of suspicion for kidney stones even in the absence of typical signs and symptoms. A diagnostic algorithm should be proposed for appropriate diagnosis and management of recurrent UTIs with further imaging in the older population to prevent recurrent admissions, debility, morbidity, and mortality. 

## Conclusions

Diagnosis of nephrolithiasis is challenging in older adults especially those with dementia due to an asymptomatic or atypical presentation. Patients with dementia are at an increased risk of poor oral intake and dehydration and therefore have a higher risk of developing kidney stones. This case report highlights the importance of further investigation for kidney stones in older adults presenting with recurrent UTIs with risk factors of dehydration who are unable to provide a reliable history and physical exam. The formation of a diagnostic algorithm is necessary to help healthcare providers determine the underlying cause of recurrent UTIs such as kidney stones in older adults with dementia. This will help to prevent morbidity, mortality, and unnecessary hospitalizations in this complex and frail population.
